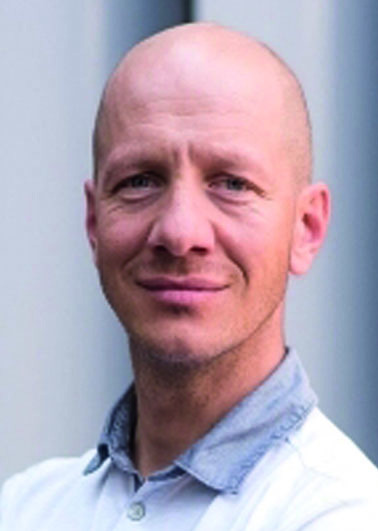# Microfluidics for Electrochemical Energy Conversion and Storage: Prospects Toward Sustainable Ammonia Production

**DOI:** 10.1002/tcr.202400234

**Published:** 2025-02-05

**Authors:** Ervin Rems, Ana Herceg, Desislava Yordanova Apostolova, Robert Dominko, Primož Jovanovič, Bostjan Genorio

**Affiliations:** ^1^ Department of Materials Chemistry National Institute of Chemistry Hajdrihova 19 1001 Ljubljana Slovenia; ^2^ Faculty of Chemistry and Chemical Technology University of Ljubljana Večna pot 113 1000 Ljubljana Slovenia; ^3^ Alistore-European Research Institute CNRS FR 3104 Hub de l'Energie Rue Baudelocque 80039 Amiens France

**Keywords:** Ammonia, Electrochemistry, Energy conversion, Microreactors, Nitrogen reduction reaction

## Abstract

Ammonia is a key chemical in the production of fertilizers, refrigeration and an emerging hydrogen‐carrying fuel. However, the Haber‐Bosch process, the industrial standard for centralized ammonia production, is energy‐intensive and indirectly generates significant carbon dioxide emissions. Electrochemical nitrogen reduction offers a promising alternative for green ammonia production. Yet, current reaction rates remain well below economically feasible targets. This work examines the application of electrochemical microfluidics for the enhancement of the rates of electrochemical ammonia synthesis. The review is built on the introduction to electrochemical microfluidics, corresponding cell designs, and the main applications of microfluidics in electrochemical energy conversion/storage. Based on recent advances in electrochemical ammonia synthesis, with an emphasis on the critical role of robust experimental controls, electrochemical microfluidics represents a promising route to environmentally friendly, on‐site and on‐demand ammonia production. This review aims to bridge the knowledge gap between the disciplines of electrochemistry and microfluidics and promote interdisciplinary understanding and innovation in this transformative field.

## Graphical abstract

This review provides an overview of electrochemical microfluidics and its emerging applications in energy conversion and storage. The focus is on the potential of the microfluidic regime for green and on‐site ammonia production by electrochemical nitrogen reduction.

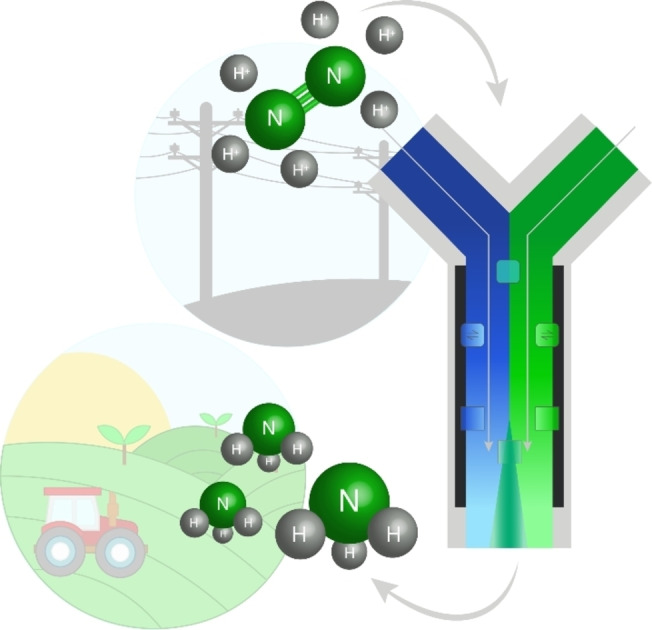

## 1 Introduction

**Figure 1:**
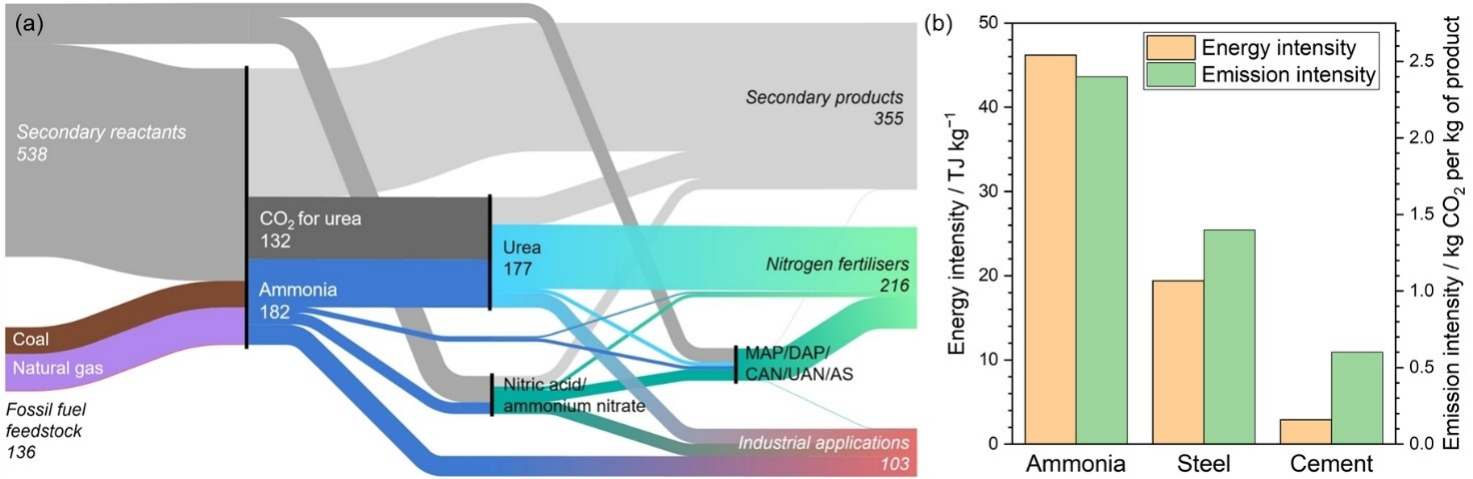
The role of ammonia in the global economy: (a) Mass flows in the ammonia supply. Numerical values are given in Tg (2019 data). MAP, DAP, CAN, UAN, and AS are monoammonium phosphate, diammonium phosphate, calcium ammonium nitrate, urea ammonium nitrate, and ammonium sulfate, respectively. (b) Energy and emission intensities for key industrial products (2021 data). Adapted from Ref. [1], International Energy Agency, CC BY 4.0.

Humanity produces more than 150 teragrams of ammonia annually, making ammonia the second most‐produced chemical globally. 70 % of ammonia is used in the synthesis of fertilizers, while the rest is used in various industrial applications, e. g., refrigeration, nitric acid production, plastics, dyes, textiles, and explosives.[^1^, ^2^] Ammonia is also emerging as a promising hydrogen‐carrying fuel.^[3]^ At the same time, ammonia production is responsible for 2 % of global energy consumption and about 1.3 % of global carbon dioxide emissions. The ammonia supply chain and energy and emission intensity of ammonia production are illustrated in Figure 1.

Industrial ammonia production relies on the Haber–Bosch process, i. e., direct synthesis from hydrogen and nitrogen in the presence of iron‐based catalysts.^[4]^ Hydrogen used in the process is produced from methane through steam reforming – water gas shift path, releasing large amounts of carbon dioxide. Additionally, high temperature (≈500 °C) and pressure (≈20 MPa) are required to ensure a suitable reaction kinetics and chemical equilibrium, respectively. This makes the process energy‐intensive, increasing cost and greenhouse gas emissions. Thus, creating a green, cost‐effective pathway for ammonia production is essential.[^5^, ^6^]

While substantial progress in the catalyst design enables Haber–Bosch synthesis at milder reaction conditions, typically at temperature above 300 °C and pressure above 0.1 MPa,^[7]^ an alternative approach employs water as a hydrogen source:^[5]^

(1) $$\begin{array}{c} {2N}_{2}+6H_{2}O\to 4{NH}_{3}+3O_{2}\# \end{array}$$

Reaction (1) – nitrogen reduction reaction (NRR) – necessitates the excitation of electrons in the reagents, which can be achieved through photochemical^[8]^ or electrochemical^[9]^ activation. The NRR is not anticipated to replace Haber‐Bosch technology.^[10]^ Instead, it aims to enable on‐site ammonia production, which would lower the transport and hazard cost and, in this way, additionally reduce carbon dioxide emissions.

The grand challenge of electrochemical ammonia production is the competing hydrogen evolution reaction (HER). Specifically, the overpotential for NRR is considerably larger than for HER, which results in poor Faradaic efficiency and ammonia formation rate in aqueous media.^[11]^ However, the recent reports on NRR in organic solvents, including ionic liquids,^[12]^ and the use of mediators^[13]^ have achieved significantly improved NRR kinetics. The improved kinetics pushes the reaction towards a mass‐transfer‐limited regime. The generally poor solubility^[14]^ and diffusivity^[15]^ of nitrogen significantly impede its transport. An interesting strategy for substantially improving the transport in devices for electrochemical energy conversion and storage is electrochemical microfluidics, i. e., the design of electrochemical cells with channels with dimensions of tens to hundreds of micrometers.[^16^, ^17^]

Here, we summarize the role of microfluidics in electrochemical energy conversion/storage and identify it as an avenue for bringing electrochemical ammonia synthesis closer to economically feasible targets. First, we introduce the basic principles of electrochemical energy storage/conversion, microfluidics, and specifics of electrochemical microfluidics and its applications. Next, state‐of‐the‐art electrochemical ammonia production is presented, and major pitfalls for its economic feasibility are identified. Finally, we discuss how electrochemical microfluidics could advance efforts in electrochemical ammonia synthesis and conclude with research directions for the field.

## 2 Microfluidic Electrochemical Cells

### 2.1 Galvanic and Electrolytic Cells

The electrochemical reaction is a chemical reaction, in which electrons are transferred between reactants through an external electronically‐conductive material:

(2) $$\begin{array}{c} C^{m+}+A^{n-}\vec{\leftarrow }C^{(m-z)+}+A^{\left(n-z\right)-}\# \end{array}$$

where z is the number of electrons transferred in the reaction

**Figure 2:**
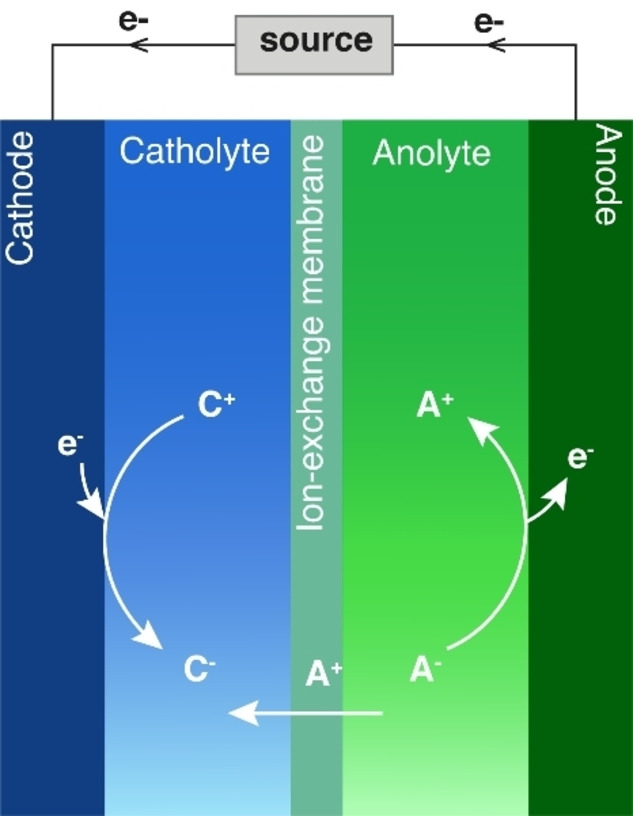
A scheme of a one‐electron electrochemical cell in an electrolytic mode. A source of direct electrical current drives a thermodynamically non‐spontaneous reduction and oxidation at the cathode and anode, respectively.

An electrochemical cell is a device that converts electrical energy into chemical energy and *vice versa* through an electrochemical reaction. The main components of a cell are cathode, anode, electrolyte, and separator (Figure 2). The electrons are exchanged between the electrodes through an external electric circuit, manifesting in an electric current. The circuit is closed through an ionically conductive electrolyte. The separator, e. g., an ion‐exchange membrane, is used to ensure ion‐specific transport within the electrolyte. Electrochemical cells, in which a spontaneous and nonspontaneous reaction occur are termed galvanic and electrolytic cells. When a spontaneous reaction occurs in the cell, it serves as a source of electrical energy. Conversely, electrical energy from an external source must be supplied to drive a nonspontaneous reaction. (Figure 2). In electrochemical energy conversion, fuel cells and electrolyzers are generally used as galvanic and electrolytic cells, respectively. Rechargeable batteries combine both modes of operation: on discharge, the battery is a galvanic cell, and on charge, it is an electrolytic cell.^[19]^

The key metric for energy conversion/storage devices is their energy efficiency η .^[20]^ Energy efficiency of an electrochemical cell is a product of Faradaic efficiency (FE) and voltage efficiency (VE):

(3) $$\begin{array}{c} \eta =\frac{useful\;energy\;input}{total\;energy\;input}=FE•VE\# \end{array}$$

Faradaic efficiency measures how efficiently the charge is utilized during a desired electrochemical process. In other words, it quantifies the selectivity of an electrochemical process.^[21]^ For a (microfluidic) electrochemical flow electrolyzer, Faradaic efficiency can be calculated as:

(4) $$\begin{array}{c} FE=\frac{q_{t}}{q}=\frac{iA}{zcF\dot{V}}\# \end{array}$$

where $q_{t}$ is the charge theoretically needed to drive the electrolytic reaction, q is the total experimental charge passed, i is the current density, A is the electrode area, c is the molar concentration, and $\dot{V}$ is the volumetric flow rate.^[17]^

Voltage efficiency describes the relationship between the thermodynamic potential difference, $E^{\circ }$ , and the experimental potential difference, E . In electrolytic cells, a potential difference, higher than the thermodynamic, must be applied to drive the electrochemical reaction. Thus, the voltage efficiency of an electrolyzer is computed as:

(5) $$\begin{array}{c} VE=\frac{E^{\circ }}{E}\# \end{array}$$

**Figure 3:**
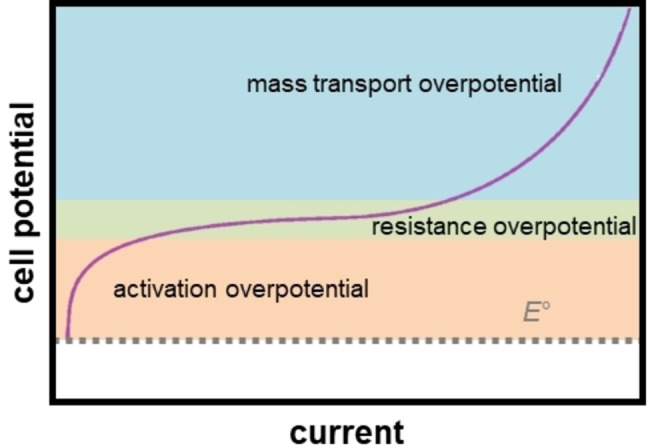
A polarization curve for an electrolytic cell.

Overpotential, i. e., the additional potential beyond the thermodynamic requirement, can be divided into contributions from activation, resistance, and mass transport (Figure 3). The activation overpotential is pronounced at low currents (slow electrochemical reaction), where the reaction kinetics is limited by the activation of electron transfer. At high currents, which correspond to fast electrochemical reaction kinetics, the mass transport overpotential prevails. This overpotential is caused by the depletion of electrochemically active species at the electrolyte–electrode interface, due to the slow transport of these species from the bulk electrolyte to the interface. Additionally, the ohmic resistance of electrodes, electrolyte, connectors, and wires results in the resistance overpotential, which increases linearly with the current (Ohm's law).

The energy efficiency of electrochemical energy conversion/storage devices increases through the minimization of overpotential. The activation overpotential is an intrinsic feature of the underlying redox chemistry and can only be improved by the identification and discovery of materials with better reaction kinetics. On the other hand, resistance and mass transport overpotentials can be minimized through careful engineering of electrochemical cells and devices.[^22^, ^23^] In this context, the microfluidic design of electrochemical cells is emerging as a powerful strategy for enhancing the energy efficiency of devices for electrochemical energy conversion/storage.[^17^, ^24^]

### 2.2 Microfluidics in Electrochemistry

Microfluidics is the science and technology of systems that process or manipulate small volumes (10^−9^ to 10^−18^ liters), using channels with dimensions of tens to hundreds of micrometers.[^16^, ^25^] The small value of the characteristic length in microfluidic systems results in low values of the Reynolds number, Re, i. e., the dimensionless number expressing the ratio between inertial and viscous forces:

(6) $$\begin{array}{c} Re=\frac{inertial\;forces}{viscous\;forces}=\frac{\rho ud}{\mu }\# \end{array}$$

where *ρ* is the density of the fluid, *u* is the flow speed, *d* is the characteristic length (the hydraulic diameter), and *μ* is the dynamic viscosity of the fluid. Low‐diameter microchannels are characterized by a Reynolds number well below the critical value (Re≪2000), indicating that the flow is governed by viscous forces, i. e., a laminar flow regime with no turbulence. Additionally, if the Reynolds number is sufficiently low (Re≪1), the microchannel adheres to the Stokes flow, i. e., flow with negligible advective inertial forces. In the Stokes flow regime, the flow of two fluids with the same viscosity and density form a stable interface with no chaotic mixing between the streams.^[26]^

In the context of electrochemical microfluidics, this opens an opportunity for membrane‐less cell architecture.[^17^, ^27^] Anolyte and catholyte streams are separated due to the Stokes flow regime. Therefore, the anolyte–catholyte interface is prone to diffusion but not advection. The ions must be exchanged at the anolyte–catholyte interface, while the fuel/product crossover must be prevented. The crossover can be controlled by tuning the Péclet number Pe, i. e., the dimensionless number expressing the ratio between the advective and diffusive transport rates:

(7) $$\begin{array}{c} Pe=\frac{advective\;transport\;rate}{diffusive\;transport\;rate}=\frac{Hu}{D}\# \end{array}$$

where *H* is the characteristic length (the electrode length), and *D* is the diffusion coefficient of the fuel/product species. Large values of the Péclet number (Pe≫1) suggests that fluid flow is strong enough to suppress significant diffusion across the channel, maintaining the integrity of the flow and effectively minimizing fuel or product crossover.^[28]^ The diffusion‐controlled mixing of the streams results in a thin diffusion zone at the center of the channel. This zone can be controlled by tuning the cell architecture parameters and the flow rate.

### 2.3 Cell Design

**Figure 4:**
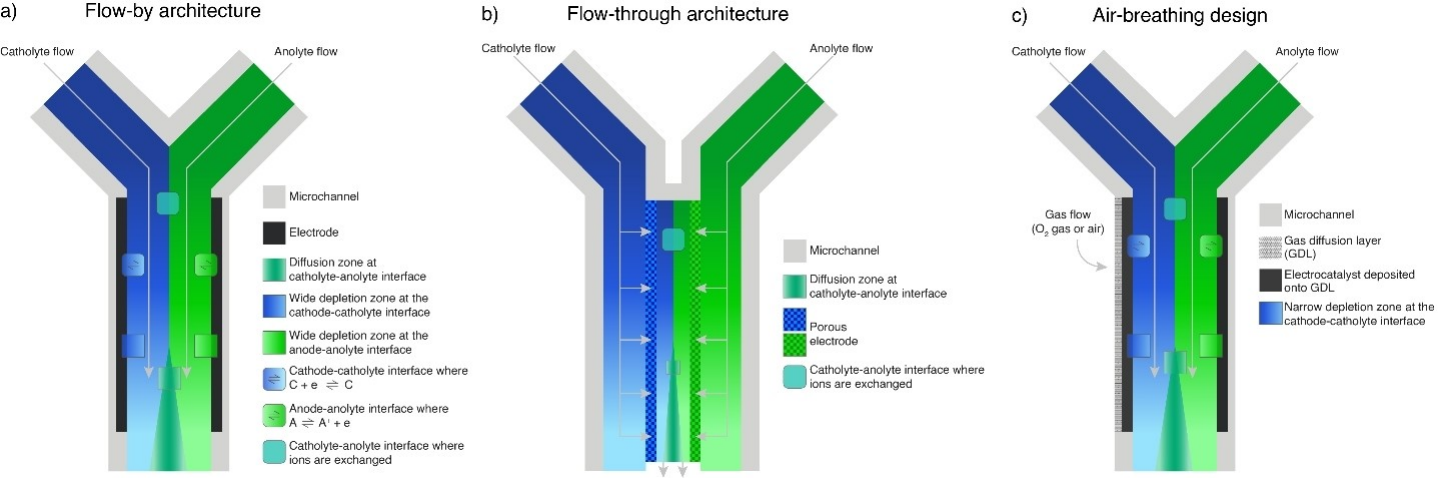
The most common designs of electrochemical microfluidic cells: (a) the flow‐by architecture is based on the horizontal flow of streams over the electrode surface; (b) the flow‐through architecture relies on stream flow through the pore network of the porous electrode; (c) the air‐breathing architecture employees a gas diffusion electrode. Schemes inspired by Ref. [47].

The architecture of the electrochemical microfluidic cell crucially affects its performance. In Figure 4, the three most often reported cell architectures are illustrated. The first reports on microfluidic cells employed flow‐by or flow‐over architectures.[^28^, ^29^] In flow‐by (Figure 4a) and flow‐over cells, the electrodes are located on the side and bottom walls of the cell, respectively. In both, the streams merge horizontally and flow over the electrode surface. The fast kinetics of the electrochemical reaction causes a concentration polarization, manifesting as a concentration boundary layer formed along the electrode–electrolyte interface. This causes a mass‐transport‐limited process, impeding the overall device performance.

A flow‐through architecture (Figure 4b) addresses the challenges posed by the concentration boundary layers formation in flow‐by and flow‐over designs and was first reported in 2008.^[30]^ Here, the use of highly porous electrodes enables the flow of the streams through the network of electrode pores. This design ensures a large, effective active area due to the high penetration of the fluid and improves the mass transport, minimizing the concentration boundary layer. Thus, the energy efficiency is improved compared to flow‐by and flow‐over designs.

Electrochemical reactions for energy conversion often rely on gases, e. g., hydrogen, carbon dioxide, oxygen, etc. The poor solubility and diffusive transport of gases in liquids, such as water, decrease the rate of such processes. An air‐breathing architecture (Figure 4c) resolves this issue by incorporating a gas diffusion electrode.^[31]^ A gas diffusion electrode is a porous, high‐surface‐area electrode in which an electrocatalyst is deposited onto a gas diffusion layer.^[32]^ Thus, the gaseous reactant can diffuse directly through the gas diffusion layer and react as soon as it dissolves in the electrolyte, eliminating the concentration boundary layer.

### 2.4 Applications

**Figure 5:**
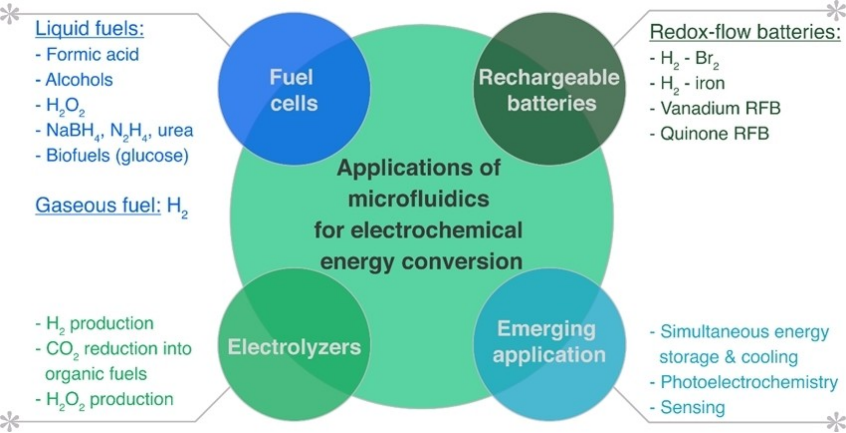
Graphical summary of applications in all areas of electrochemical microfluidics: electrolyzers, fuel cells, and redox flow batteries.

The paramount applications of electrochemical microfluidics were reported in the early 2000s as membrane‐less fuel cells based on laminar flow. These fuel cells were based on liquid fuels, i. e., formic acid^[28]^ and vanadium‐based redox pairs.^[29]^ Since then, many microfluidic fuel cells based on different chemistries, e. g., hydrogen,^[33]^ alcohols,^[34]^ sodium tetrahydroborate,^[35]^ hydrogen peroxide,^[36]^ etc., have been reported. Simultaneously, the electrochemical microfluidics expanded to electrolyzers^[37]^ and rechargeable redox flow batteries^[38]^ (Figure 5). Other applications relevant to electrochemical energy storage/conversion include microfluidics in photoelectrochemistry,^[39]^ sensing,^[40]^ and nanomaterials synthesis,^[41]^ including the synthesis of an NRR electrocatalyst.^[42]^

Microfluidic membrane‐less electrolyzers were mostly explored for hydrogen evolution[^37^, ^43^, ^44^] and carbon dioxide reduction (e. g., into formic acid,^[45]^ carbon monoxide,^[46]^ and methanol).^[47]^ It has been shown that the performance of parallel plate membrane‐less water electrolyzers can exceed that of alkaline electrolyzers in key performance metrics (energy efficiency, current density). However, they remain inferior to polymer electrolyte membrane electrolyzers.^[48]^ Similarly, microfluidic electrolyzers were considered for chlorine and sodium hydroxide production through the chloralkali process,^[49]^ hydrogen peroxide,[^50^, ^51^] and sodium hypochlorite.^[52]^ Inspired by the rise of microfluidic electrolyzers, we critically assess whether a microfluidic regime of ammonia electrosynthesis could bring the ammonia formation rates closer to target values of commercial viability.

## 3 Electrochemical Microfluidics for Ammonia Production

### 3.1 Electrochemical Nitrogen Reduction

Electrochemical ammonia synthesis is defined by

(8) $$\begin{array}{c} N_{2}\left(g\right)+8H^{+}\left(aq\right)+6e^{-}\vec{\leftarrow }2{NH}_{4}^{+}\left(aq\right)\;\# \end{array}$$

with the standard reduction potential equaling *E°*=0.334 V vs RHE (pH=1).^[53]^ To minimize capital and operational costs, a nitrogen reduction electrocatalyst must perform at high and stable current densities, high Faradaic efficiency, and low overpotential. The US Department of Energy indicates that an efficient nitrogen reduction electrocatalyst must provide 10^−8^ mols^−1^ cm^−2^_geo_ and 90 % Faradaic efficiency. However, state‐of‐the‐art technologies are still far short of this target.^[6]^ The low Faradaic efficiencies may be attributed to the competing hydrogen evolution reaction (HER) with standard reduction potential similar to nitrogen reduction but much simpler and faster kinetics.^[53]^

Conventionally, NRR has been considered as an associative (hydrogenation of N_2_ via a distal or alternating path) or a dissociative mechanism (N_2_ and H_2_ do not react until the strong N_2_ triple bond and the H_2_ bond have been broken, Haber‐Bosch case). Both pathways might proceed via a Tafel‐type mechanism (i. e., adsorbed hydrogen adatoms react with adsorbed N_2_H_x_ or NH_x_ species). Alternatively, a Heyrovsky‐type mechanism is possible (adsorbed N_2_H_x_ or NH_x_ species undergo hydrogenation via direct protonation from the solution coupled with electron transfer). However, considering the substantially higher activation barrier for the Tafel‐based mechanism, NRR is expected to proceed via the Heyrovsky path. Regarding the distinction between the associative and dissociative pathways, the former is considered viable on early transition metals (Sc, Y, Ti, Zr) as N‐based intermediates bind stronger than H‐adatoms, hypothesizing the prevalence of NH_3_ formation over HER.^[54]^ A dissociative path, on the other hand, was demonstrated to proceed on a flat and stepped Ru(0001) surface.[^55^, ^56^, ^57^]

Notably, for these mechanisms, the nitrogen and hydrogen binding energies generally scale linearly, meaning that one cannot manipulate the nitrogen binding energy without influencing the hydrogen binding energy. Therefore, improving activity towards NRR is exceptionally challenging since potential catalysts would need to circumvent the nitrogen‐hydrogen scaling relations. As shown from computational predictions, proceeding through a different mechanism could change the binding energies of the intermediates independently. In this respect, a Mars‐van Krevelen mechanism has been considered as well.^[58]^ In this case, the catalytic cycle involves the insertion of a species (e. g., metal nitrides) into the catalyst layers, where a nitrogen‐containing composite has nitrogen hydrogenated to form NH_3_. The latter then desorbs from the catalyst structure as the first NH_3_ molecule. Subsequently, N_2_ can be inserted into the catalyst structure where one of the nitrogen atoms can be incorporated into the catalyst structure, whereas the second is consumed to produce the second NH_3_ molecule. However, we emphasize that this particular mechanism, along with the activity of the catalysts, might be considered speculative since the catalyst breakdown, in fact, results in significant amounts of evolved NH_3_.[^59^, ^60^]

A plethora of different structural motifs have been reported with claimed NRR activity, including noble metals,[^61^, ^62^] metal oxides,[^63^, ^64^] metal sulfides,^[65]^ metal nitrides,[^66^, ^67^] non‐metallic catalysts,[^68^, ^69^] and, in particular, single atoms.[^70^, ^71^, ^72^, ^73^, ^74^, ^75^] However, due to insufficient measures in N_2_ quantification (see below), the NRR performances are ambiguous.

Noteworthy, a considerable success in NRR has also been achieved through indirect, non‐aqueous lithium‐mediated NRR pathway.[^76^, ^77^, ^78^, ^79^] Here, electrodeposition of Li^+^ to Li metal is performed first. Li metal then reacts with nitrogen to form Li_3_N intermediate, which can accept protons to form NH_3_ and simultaneously regenerate Li^+^. Li‐mediated NNR has been reported in both batch[^80^, ^81^] and continuous[^82^, ^83^] setups. Li‐mediated NRR is beyond the scope of this review, and the reader is referred to existing reviews on the topic.[^13^, ^84^, ^85^, ^86^, ^87^]

### 3.2 The Importance of Experimental Controls in Electrochemical Ammonia Synthesis

Before critically examining the potential relevance of microfluidics, it is crucial to consider the skepticism expressed by experts in the field, particularly the lack of strict experimental controls. Namely, to conduct a credible NRR investigation, one should initially minimize potential contamination sources, especially nitrate, nitrite, and NH_3_ in the N_2_ stream. The direct method to prove the electrochemical origin of NH_3_ is the ^15^N_2_ labeling experiment. One should keep in mind that, despite numerous groups reported on the ^15^N_2_ labeling experiment, the experiment might give unreliable results as ^15^N_2_ gas contamination is frequently overlooked. Specifically, the pretreatment of ^15^N_2_, along with adequate background control, is rarely performed to ensure that no ^15^N containing NH_3_ and NO_x_ are present in the ^15^N_2_ itself. In addition, in most NRR reports, ^15^N_2_ controlled experiments are conducted solely as a qualitative analysis, while they should also be demonstrated catalytically. Therefore, if proper ^15^N labeling protocols are followed by the achieved NRR rate of more than 10^−8^ mols^−1^ cm^−2^_(geom)_, such a study can be considered credible.^[88]^ A very important, yet rarely engaged, practice is reporting the NRR performance metrics carefully. Ideally, NRR performance (apart from the FE parameter) should be given as a specific current density (i. e., current per active surface area *j*_NH3real_).^[89]^ We note, however, that for a vast majority of reported catalysts, the accurate determination of the electrochemically active surface area (ECSA) is not straightforward. Alternatively, *j*_NH3geo_ can be used, but in this case, one should critically assess the catalyst loading.

### 3.3 State‐Of‐The‐Art

**Figure 6:**
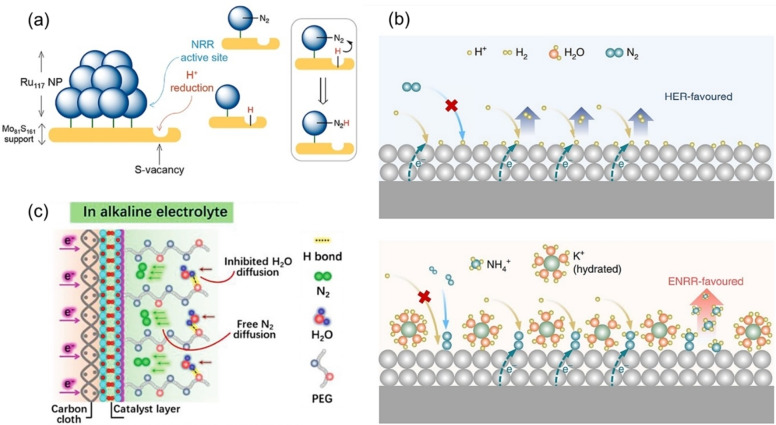
Strategies for optimization of proton source concentration at the interface: (a) modulation of conductivity of the electrode substrate to increase the HER overpotential, (b) electrolyte design for hindered proton transfer from bulk to the interface, (c) suppressing proton diffusion to the interface through hydrogen bonding between an additive and proton source. Reprinted with permission from: (a) Ref. [99]. Copyright 2019 American Chemical Society, (b) Ref. [93]. Copyright 2019, The Authors, under exclusive license to Springer Nature Limited, (c) Ref. [98]. Copyright 2021, Wiley‐VCH GmbH.

As noted above, circumventing HER is a crucial requirement for a successful NRR electrocatalyst. Currently, there are only a handful of cases (even from those considering the studies following unambiguous NH_3_ quantification) capable of reaching *j*_NH3geo_ of 10^−8^ mols^−1^ cm^−2^_geo_.[^65^, ^90^, ^91^, ^92^, ^93^] Furthermore, none satisfy the performance thresholds for NRR electrocatalysts for profitable uptake and implementation.^[94]^ The performances (here referred to as *j*_NH3geo_) of a vast majority of aqueous‐based systems are at least two orders of magnitude lower than the set target. Alternatively, to tackle the HER, it would be reasonable to transfer the NRR platform into an aprotic media such as aprotic solvents and/or ionic liquid‐based electrolytes, as demonstrated recently.[^80^, ^95^, ^96^, ^97^, ^98^] Accordingly, high FEs can be obtained, whereas the *j*_NH3geom_ values are around 10^−11^ mols^−1^ cm^−2^_geo_,^[95]^ which is still substantially lower than the aqueous record‐breakers. Of note here is that, typically, the NRR catalysts from the non‐aqueous platform are rarely further optimized. There is considerable scope for their further development and optimization of other parameters, such as electrode composition and structure. Regardless of particular conditions, the grand challenge is an optimized proton source concentration for the NRR process. Namely, if it is too high, the HER begins to dominate, whereas if it is too low, the proton supply becomes limiting to the rate of NRR. Here, the most promising strategies either focus on optimizing the NRR composite or the electrolyte (Figure 6):

- The selectivity toward NRR can be increased by manipulating the conductivity of the electrode substrate and increasing the HER overpotential (Figure 6a). This has recently been shown to be possible on a composite based on semiconducting MoS_2_ support (serving as H binding sites) decorated with Ru clusters (serving as N_2_ binding sites).^[99]^
- By slowing proton transfer from the bulk solution to the electrode surface, proton migration from the bulk solution to the electrode surface can be inhibited by high‐concentration supporting electrolytes via cation‐induced interactions. Namely, highly concentrated cations in the diffusion layer at low potentials would hinder proton transfer down to the rate‐limiting regime. This has been demonstrated in the case of K^+^ cations (Figure 6b), resulting in impressive FEs and NRR current densities (in the range of 10^−8^ mols^−1^ cm^−2^_geo_).^[93]^ However, we note that the ^15^ N labeling verification was lacking.^[100]^ Additionally, high electrolyte concentrations can influence proton availability via ion solvation by weakening the H_2_O−H_2_O interaction and strengthening the H_2_O–ion interaction. This results in a substantial reduction of free H_2_O molecules, hence inhibiting HER as demonstrated via Li^+^ cations.^[101]^
- Alternatively, HER can be retarded by implementing task‐specific electrolyte additives. Here, the so‐called molecular crowding effect enabling reduced proton diffusion via hydrogen bonding between the additive and the proton source (not affecting the transport of N_2_, though) stands out and has been demonstrated via the implementation of poly(ethylene glycol) (Figure 6c).^[98]^

The mentioned studies demonstrate the relevance of the NRR reaction interface where, as long as the proton content and the HER kinetic/proton transport can be well balanced, high‐performance NRR might also be possible. This certainly provides credible motivation for the implementation of the microfluidic regime. Particularly concerning the control of N_2_ and proton access, as discussed in detail later.

### 3.4 En Route to Microfluidics

Before considering the potential relevance of the microfluidic regime, one needs to critically assess the state‐of‐the‐art NRR electrocatalysts in the context of mass transport limitations, which are scarcely discussed in the literature. Herein, we refer to a theoretical estimation of mass transport limitation imposed by N_2_ diffusion previously derived by Lazouski et al.^[102]^ We emphasize that the mentioned work considered a non‐aqueous (non‐aqueous lithium mediated approach) NRR case. However, in this particular case, the NRR kinetics can, indeed, reach sufficiently high rates, leading to N_2_ mass transfer becoming the rate‐limiting factor.[^77^, ^78^, ^103^, ^104^] Accordingly, the limiting current density for NRR would be 12 mA/cm^2^_geom_±8 mA/cm^2^_geom_ (at FE=100 %) under stagnant conditions. Under an aqueous regime, a lower value is expected due to lower N_2_ solubility.[^96^, ^105^] Regardless, the above criteria signify that state‐of‐the‐art electrocatalysts are far from operating under full mass transport control (i. e., several orders of magnitude lower *j*_NH3geo_ reported). Interestingly, it is yet unclear whether electrochemical configurations enabling elevated mass transport of N_2_ capitalizing from the triple‐phase contacting (e. g., GDE, MEA as widely deployed in fuel cells and electrolyzers for O_2_ and CO_2_ reduction) can perform better than liquid‐based cells (i. e., operating via liquid‐liquid contacting).^[106]^ This might be explained through theoretical calculations, which suggest that N_2_−H_2_O electronic interactions cause a lower kinetic barrier in contrast to the N_2_ gas/solid catalyst analogy.^[107]^

**Figure 7:**
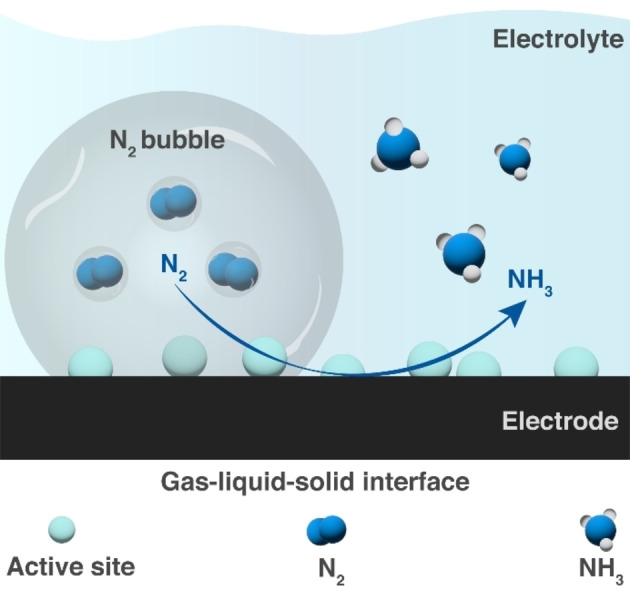
Scheme of the NRR at the triphasic N_2_‐electrolyte‐electrode interface in the bubble‐based microreactor, adapted and modified based on Ref. [104].

However, the work of Wei et al., based on implementing a GDE‐based configuration, provides a solid motivation for the triple‐phase interface.^[101]^ In particular, a microtubular GDE geometry was used, enabling N_2_ supply towards the triple‐phase boundary, resulting in promising *j*_NH3geo_ of 10^−9^ mols^−1^ cm^−2^_geo_ range. Crucially, this was an order of magnitude higher than the result observed when N_2_ was supplied by bubbling the electrolyte, strongly implying that the NRR can be susceptible to mass transport limitations. Further relevant work exploiting the triple‐phase contacting was introduced by Liu et al., implementing a microfluidic‐based configuration.^[108]^ More specifically, the N_2_ was supplied to the electrode via microbubbles, meaning that a three‐phase‐based regime is operative since the bubbles attached to the electrode surface represent highly abundant three‐phase junctions (Figure 7). Remarkably, the microreactor demonstrated substantially better performance than fully flooded configurations, giving further credence to the N_2_ mass transport enhancement. Drawing from this sole example of microfluidics applied to NRR, combined with the knowledge from general microfluidic practice, the NRR performance could be enhanced by exploiting the microfluidic regime.

Undivided cells, often used in NRR for simplicity, suffer from NH_3_ and NH_4_^+^ crossover by diffusion or convection with minimal restriction, leading to undesirable product oxidation. Often overlooked, product oxidation may result in underestimating the actual process performance. In an attempt to mitigate product crossover, various membranes were tested. However, cation‐exchange membranes (Nafion®) accumulated NH_4_^+^ product, leading to membrane degradation, losing the capability to impede NH_4_^+^ transport, and discharging NH_4_^+^ into the anode compartment. The only membrane not absorbing significant NH_4_^+^ amounts was the Celgard® membrane in neutral media. Regardless, the problems concerning ohmic resistance and membrane reactor maintenance remain present.[^106^, ^109^] These issues can be solved by implementing microfluidics. Due to the small interelectrode gap and the absence of a membrane, microreactors reduce the resistance to ion migration in the electrolyte while preventing product oxidation concurrently, thanks to the laminar flow. More precisely, the laminar flow, developed within a microchannel, prevents catholyte and anolyte mixing, thus preventing product oxidation.^[110]^ Notably, since microreactors mitigate membrane usage, electrolytes with various pH values can be implemented. Moreover, microreactors offer the possibility of dual‐electrolyte operation, which enables tailoring pH conditions each half‐cell benefits from, minimizing the total cell voltage.^[17]^

Finally, it is crucial to highlight the importance of real‐time monitoring to gain insight into the processes occurring within a microreactor. Inline detection using appropriate sensors emerged as the most robust method to monitor reaction conditions, reaction progression, and fluctuations indicating system instability. Therefore, inline detection enables swift corrective action, enhancing process control, reducing waste, and improving product quality.[^111^, ^112^] A successful example is presented in H_2_O_2_ production, where an optical sensor was developed that did not alter the electrolyte's properties, enabling its reuse.^[113]^ Here, we advise developing a similar sensing system for ammonia detection.

## 4 Summary and Outlook

We highlighted the pivotal benefits of the NRR in a microfluidic setup: enhanced mass transport, lower ohmic resistance, and better control over the electrochemical process. Moreover, microfluidic designs offer the potential for on‐site, on‐demand production of ammonia, which can be scaled through numbering‐up strategies. Importantly, experiments could reveal additional benefits of a microfluidic regime for electrochemical ammonia production that we are unaware of.

Electrochemical reduction of carbon dioxide has already been advanced by exploiting the benefits of microfluidics.[^114^, ^115^] Given the similarities between the requirements of the NRR and CO_2_ reduction reactions, best practices in microfluidic CO_2_ reduction could provide valuable insights toward microfluidic NRR. Finally, the realization of the microfluidic NRR shall be made possible through closely collaborative, systematic, well‐controlled experimentation, multiscale physical modeling, and data‐driven approaches.

## Biographical Information

*Ervin Rems is a junior researcher at the National Institute of Chemistry, Slovenia, and a PhD student in Chemical Sciences at the University of Ljubljana, Slovenia. He obtained his BSc in Chemistry from the University of Ljubljana, Slovenia. Rems pursued a joint masters in Materials for Energy Storage and Conversion, obtaining MSc in Materials Science and Engineering from Université Toulouse III ‐ Paul Sabatier, France, and MSc in Chemistry from Université de Picardie Jules Verne, France. His research focuses on atomistic modeling of materials for energy applications*.

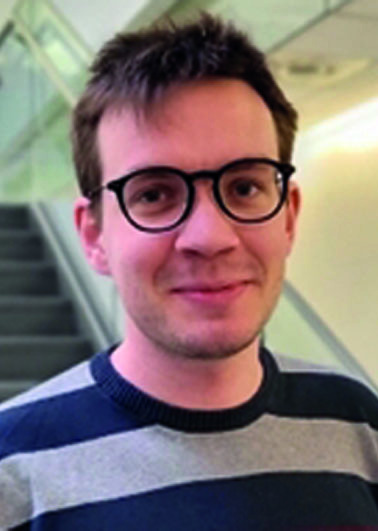

## Biographical Information

*Ana Herceg is PhD student at the University of Ljubljana, Slovenia, and a member of the Laboratory for Electrocatalysis at the National Institute of Chemistry, Slovenia. Herceg obtained her BSc and MSc in Chemical Engineering (Materials) from the University of Split, Croatia, in 2021 and 2023, respectively. Her research focuses on lithium‐mediated nitrogen reduction reaction*.

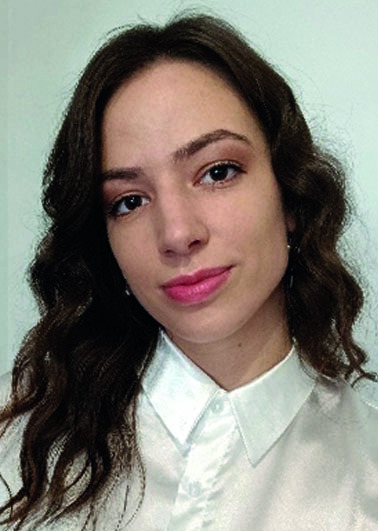

## Biographical Information

*Desislava Yordanova Apostolova is a PhD student in Chemical Sciences at the University of Ljubljana, Slovenia. She obtained her Bachelor of Science in Medicinal Chemistry from South‐West University “Neofit Rilski” in Blagoevgrad, Bulgaria. Apostolova completed her Master of Science in Innovative Technologies for Renewable Energy as part of a joint program between South‐West University “Neofit Rilski” and the University of Chemical Technology and Metallurgy in Sofia, Bulgaria. Her research focuses on the development and analysis of carbon‐based electrocatalysts and their integration into microfluidic systems*.

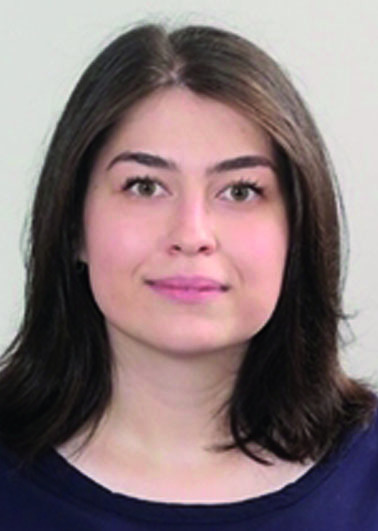

## Biographical Information

*Robert Dominko is a research professor at the National Institute of Chemistry, Slovenia, and a professor of Material Science at the University of Ljubljana, Slovenia. His research interests are materials science and electrochemical systems for energy storage, with main activities in the field of modern battery systems. His research interests are focused on lithium and multivalent batteries, and the implementation of smart functionalities in battery cells. He is a deputy director of the European virtual research laboratory for batteries Alistore ERI and a member of the Slovenian Academy of Engineering*.

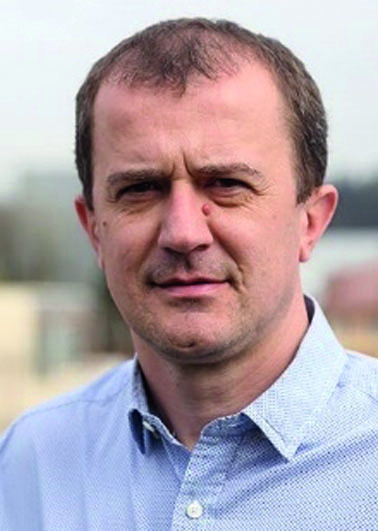

## Biographical Information

*Primož Jovanovič is a research assistant professor at the National Institute of Chemistry (NIC), Slovenia. Jovanovič completed his PhD in 2016 at NIC. Afterwards, he has been working as a postdoctoral researcher at the Laboratory for Electrocatalysis, NIC, and as a postdoctoral researcher in Freunberger Group at the Institute of Science and Technology Austria. His research interests encompass electrochemical energy conversion, electrosynthesis, and electrochemical analysis*.

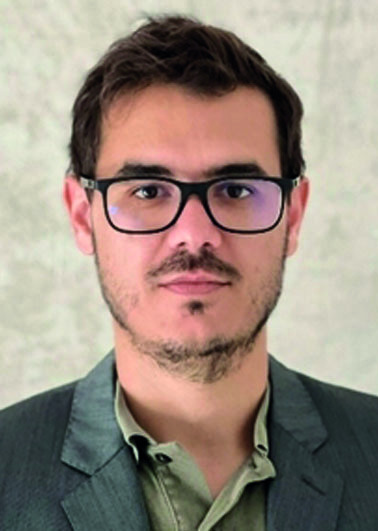

## Biographical Information

*Boštjan Genorio is an associate professor of Materials Science at the University of Ljubljana, Slovenia. He earned his BSc and PhD degrees from the University of Ljubljana. From 2011–2012, he was a postdoctoral research associate under the mentorship of James M. Tour at Rice University, Houston, Texas. From 2013–2015, he served as a visiting scientist in the Materials Science Division at Argonne National Laboratory. Genorio's research specializes in the synthesis and characterization of advanced nanomaterials for energy storage and conversion. His work has contributed to advancements in sustainable technologies, particularly in hydrogen technologies, next‐generation energy systems, and the electrosynthesis of industrial chemicals*.